# Enhancing dementia prediction: A 19-year validation of the CAIDE risk score with insulin resistance and APOE ε4 integration in a population-based cohort

**DOI:** 10.1016/j.tjpad.2024.100034

**Published:** 2025-01-01

**Authors:** Elina Pietilä, Eliisa Löyttyniemi, Seppo Koskinen, Jenni Lehtisalo, Matti Viitanen, Juha O. Rinne, Antti Jula, Laura L. Ekblad

**Affiliations:** a Turku PET Centre, University of Turku, Turku, Finland; b Turku PET Centre, Turku University Hospital, Turku, Finland; c Department of Biostatistics, University of Turku and Turku University Hospital, Finland; d Finnish Institute for Health and Welfare, Helsinki, Finland; e Department of Geriatrics, Turku University Hospital, Wellbeing services county of Southwestern Finland, Finland; f Division of Clinical Geriatrics, NVS, Karolinska Institutet, Stockholm, Sweden; g InFLAMES Research Flagship Center, University of Turku, Turku, Finland; h Finnish Institute for Health and Welfare, Turku, Finland

**Keywords:** Risk score, Dementia, Memory disorder, Insulin resistance, HOMA-IR

## Abstract

**Background:**

Dementia is a significant cause of disability and dependency. Persons with high dementia risk but intact cognition will benefit from preventive interventions.

**Objectives:**

The aim was to validate dementia risk score Cardiovascular Risk Factors, Aging and Incidence of Dementia (CAIDE) in a national population-based cohort with data on age, education, hypertension, obesity, hyperlipidemia and physical activity. Secondly, we examined if substituting obesity item with Homeostatic Model Assessment for Insulin Resistance (HOMA-IR) would improve predictive value of CAIDE risk score.

**Design:**

Longitudinal, population-based cohort study.

**Setting:**

General population, Finland

**Participants:**

Representative sample of Finnish adult population aged over 30 years from Health 2000 Survey (*n* = 5,806).

**Measurements:**

CAIDE dementia risk score and substituting BMI with HOMA-IR.

**Results:**

Dementia was diagnosed in 571 (9.8 %) participants during the 19 years follow-up. CAIDE risk score predicted dementia well: AUC (area under curve) ROC (receiver-operating characteristic) was 0.78 (95 % CI from 0.76 to 0.79). Secondly, replacing obesity with HOMA-IR in CAIDE risk score generated similar results: ROC AUC 0.78 (95 % CI from 0.76 to 0.80). Adding *APOE* ε4 status further improved predictive value of risk score: ROC AUC 0.81 (95 % CI from 0.80 to 0.83).

**Conclusions:**

CAIDE dementia risk score predicts dementia well in a national population-based cohort. Adding *APOE* ε4 genotype improved predictive value of risk score. Insulin resistance measured by HOMA-IR is comparable to obesity as part of CAIDE risk score. These findings imply that CAIDE risk score is applicable for assessing risk of dementia and highlight importance of modifiable risk factors of dementia.

## Highlights

• CAIDE dementia risk score predicts dementia well in a population-based cohort.
• Insulin resistance (HOMA-IR) is comparable to obesity as part of CAIDE risk score.
• CAIDE risk score is applicable for assessing risk of dementia.

## Graphical abstract

**Figure:**
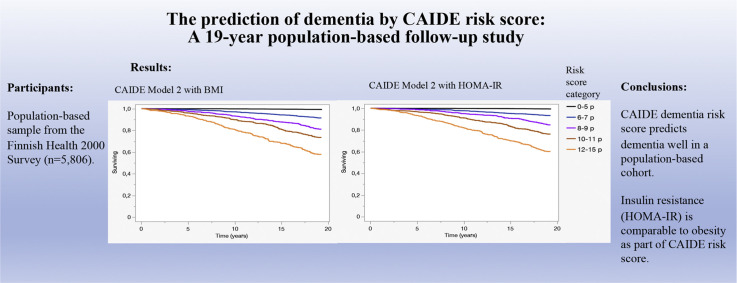

## 1 Introduction

Dementia is a significant cause of disability and dependency worldwide. Furthermore, dementia is indicated to be the seventh leading cause of death globally, underscoring the importance of this public health problem [1]. Encouragingly, findings from randomized controlled studies such as the Finnish Geriatric Intervention Study to Prevent Cognitive Impairment and Disability (FINGER) suggest that cognitively healthy persons with high risk of cognitive decline might benefit from multidomain lifestyle interventions to prevent cognitive impairment [2]. However, identifying these at-risk individuals is challenging, as the duration of the prodromal phase for dementia is very long [3].

Based on the accumulating evidence that midlife modifiable risk factors increase the risk of cognitive decline and late-life dementia [4], [5], [6] various risk scores have been developed to evaluate the cumulative impact of potentially modifiable risk factors of dementia [7], [8], [9], [10] for identification of individuals at high risk of dementia and to increase the awareness of the importance of the modifiable risk factors of dementia. It is known that *APOE* ε4 risk allele is associated with a 3 to 4 fold risk of developing Alzheimer's disease [3].

The Cardiovascular Risk Factors, Aging and Incidence of Dementia (CAIDE) score was developed to estimate late-life dementia risk during 20 years of follow-up based on midlife risk factors. It was based on a Finnish population cohort aged 39–64 years (*n* = 1409) [9]. The CAIDE dementia prediction score has been externally validated in dementia with a C statistic of 0.75 [11], which was quite similar to the original CAIDE C statistic of 0.78. The CAIDE risk score also seems to perform well among different ethnic groups [11]. A recent study has shown that a high CAIDE score even in the presence of AD pathology is associated with an increased risk of dementia in MCI subjects [12]. However, some other studies have not found an association between the CAIDE risk score and dementia risk [13], [14], [15], [16]. These studies have not been based on national cohorts and they either had shorter follow-up times [13,15,16] or the populations were relatively small [14,15]. Accordingly, there is a knowledge gap in existing studies to combine a longitudinal, population-based and a large cohort with a long follow-up.

Moreover, in addition to the CAIDE risk score being the most widely utilized risk score to predict dementia, the CAIDE dementia risk score has also been utilized in studies examining diverse outcomes such as cognitive decline [17,18], dementia-related brain changes on magnetic resonance imaging (MRI) [19,20], and the neuropathological hallmarks of Alzheimer´s disease (AD) and vascular disease [15]. Importantly, the CAIDE score was also used to select participants for the FINGER trial, a landmark clinical trial in the field of preventing memory disorders [2].

Despite the positive results of the FINGER trial i.e. greater improvement in cognitive function in the multidomain intervention group when compared to the “standard care” group during a follow-up of two years, it is still unclear which pathophysiological mechanisms are responsible for the association between vascular risk factors and cognitive decline and whether midlife vascular risk factors associate with AD neuropathology. The CAIDE risk score includes vascular risk factors such as obesity, hypercholesterolemia, smoking and elevated systolic blood pressure. However, recent research suggests that insulin resistance, closely associated with obesity and the metabolic syndrome, may play an important role in the development of AD [21], [22], [23]. For example, it has been demonstrated that in *post mortem* brain slices of AD patients the response to insulin incubation is attenuated when compared to individuals who were cognitively normal during life, suggesting that central insulin resistance exists in the AD brain [24]. Moreover, insulin and beta-amyloid, a hallmark of AD neuropathology, share a common degrading enzyme in the brain (IDE, insulin degrading enzyme). This enzyme seems to be down-regulated in insulin resistance, which could contribute to the accumulation of beta-amyloid in the brain [25]. According to these findings, also the FINGER study group has now commenced a randomized clinical trial where the effect of metformin, a drug targeted at lowering insulin resistance, on cognitive function will be evaluated in addition to the FINGER multidomain intervention (MET-FINGER, NCT05109169, clinicaltrials.gov).

The aim of this study is to validate the CAIDE dementia risk score in the Health 2000 Survey, a nationally representative sample of the Finnish adult population enhancing the generalizability of the results. Moreover, based on previous research, as both HOMA-IR [21] and elevated BMI [26] at late-midlife have been linked to brain amyloid-beta (Aβ) accumulation, which is considered to be the earliest pathological hallmark of AD, we examined if substituting the obesity variable with a measure of insulin resistance would improve the predictive value of CAIDE risk score. Insulin resistance was measured with Homeostatic Model Assessment for Insulin Resistance (HOMA-IR).

## 2 Methods

### 2.1 *Health 2000 study*

The data for this study were acquired from the Finnish population-based Health 2000 Examination Survey. The Health 2000 Survey is a representative and nationwide health examination survey of the Finnish adult population, conducted by the Finnish Institute for Health and Welfare and carried out in the years 2000–2001. In the Health 2000 study 8028 individuals aged 30 or older were selected randomly from the Finnish population register with a two-stage cluster sampling design. The participation rate in the health examination proper was 84 % (*n* = 6770). The participants underwent a thorough physical examination and the health interview, and fasting venous blood samples were collected [27].

The Finnish Health 2000 study was approved by the Ethics Committee for Epidemiology and Public Health in the hospital district of Helsinki and Uusimaa, Finland. All participants gave written informed consent for participating in the study.

### 2.2 *Study population*

**Fig. 1:**
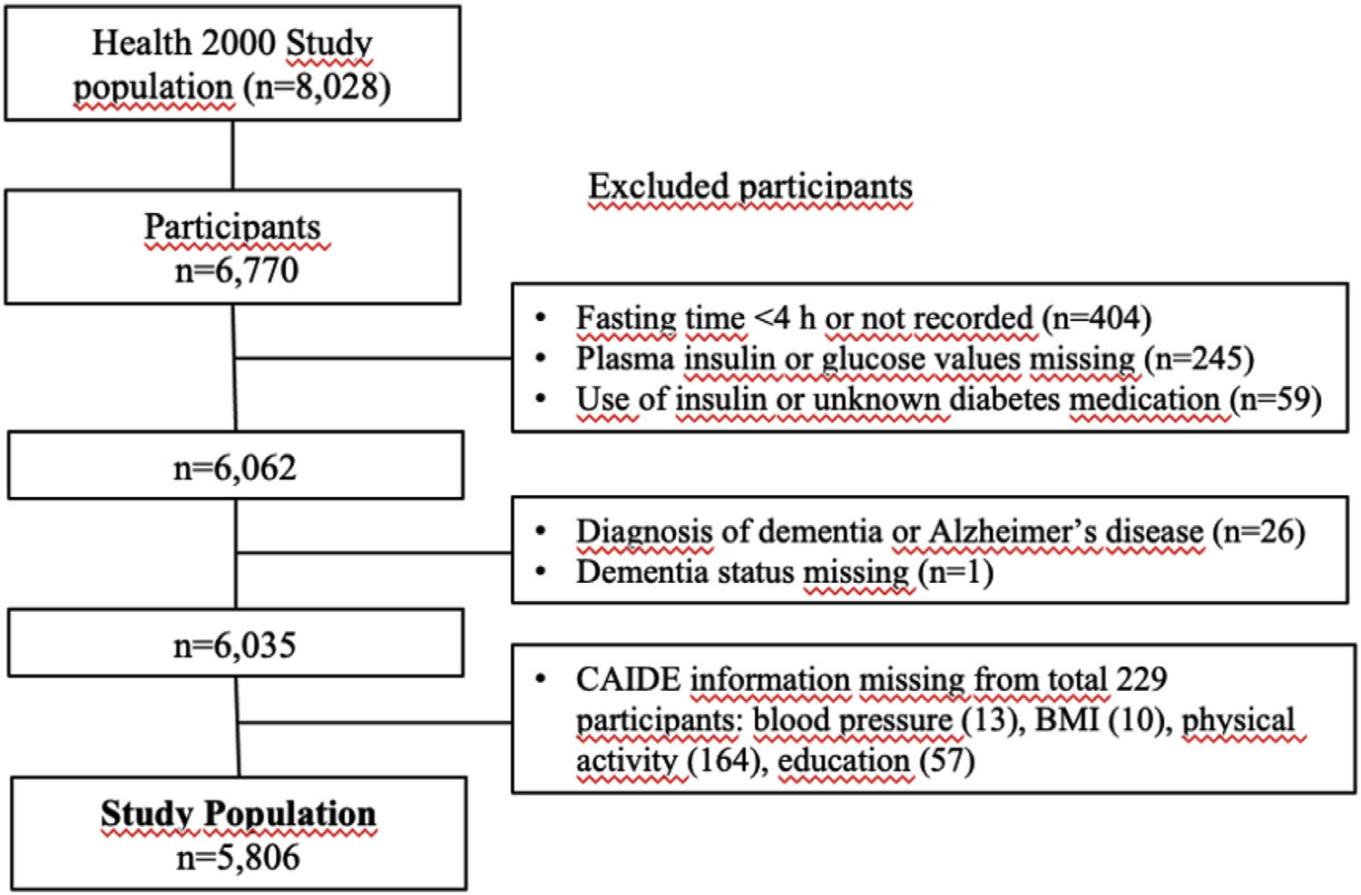
The flow chart of the study population. 6035 participants without dementia had fasting time 4 h or more and they were not using insulin. This final study population consisted of 5806 participants of whom had all the data to calculate the CAIDE risk score.

The flow chart of the study population is shown in Fig. 1. HOMA-IR values were calculated for those individuals who had fasted 4 h or more. Exclusion criteria of the study were use of insulin treatment (since HOMA-IR cannot be reliably calculated in individuals using insulin), but use of diabetes medication other than insulin was not an exclusion criterion. Exclusion criteria were also taking unknown diabetes medication, and missing plasma insulin or glucose values. Also, individuals who had diagnosis of dementia or Alzheimer's disease at the beginning of the study were excluded from calculating CAIDE dementia risk score. Altogether, the study population consisted of 5806 participants, with a mean age of 52.1 years (range 30–97); 54.8 % were females.

### 2.3 *Measurements*

Blood samples were drawn from the antecubital vein, fasting time was recorded and the samples were stored in –70 °C until they were analyzed. Cholesterol values were determined by a CHOD PAP test (Olympus System Reagent; OLYMPUS, Hamburg, Germany), HDL cholesterol values were analyzed by a HDL-C Plus test and LDL cholesterol by a LDL-C Plus test (Roche Diagnostics, Mannheim, Germany), triglycerides by a GPO PAP test (Olympus SystemReagent), and glucose values by a hexokinase test (Olympus SystemReagent). Serum insulin was determined by a microparticle enzyme immunoassay (Abbott Laboratories Dainabot, Tokyo, Japan) [28].

*APOE* ε4 genotyping was performed with the MassARRAY system (Sequenom, San Diego, California) with a modified protocol [29]. *APOE* ε4 genotype was considered positive for subjects with one or two ε4 alleles. Participants using oral diabetes medication or who had fasting glucose values > 7.0 mmol/L were classified as having type 2 diabetes. HOMA-IR was used as a measure of insulin resistance and calculated by the equation fasting insulin (mU/mL) times fasting glucose (mmol/L) divided by 22.5 [30]. Body mass index (BMI) was calculated as weight in kilograms divided by height in meters squared. Hypertension was defined as systolic blood pressure ≥ 140 mmHg or diastolic blood pressure ≥ 90 mmHg or use of antihypertensive treatment. Blood pressure was measured in a sitting position from the right arm with a standard mercury manometer (Mercuro 300; Speidel & Keller, Jungingen, Germany), and the average of two measurements was used for the analyses. Physical activity was defined as self-reported leisure time physical activity at least twice a week lasting at least 30 min and causing at least mild breathlessness and sweating. Information on education in years was collected via interviewing the participants. All the measurement data were collected in the years 2000–2001 [27].

### 2.4 ***Dementia and****Alzheimer's****disease status***

The register data of the dementia outcome was collected from Finnish national registers by the Finnish Institute for Health and Welfare. Diagnosis of dementia included ICD-10 codes F00–03 and G30 from the Care Register for Health Care and Finnish Causes of Death Register, and also data from dementia medicine purchases (N06D) or reimbursements code for dementia medicines (307) by the Social Insurance Institution of Finland. Alzheimer's disease included ICD-10 codes F00 and G30.

In Finland the diagnosis of dementia and Alzheimer's disease is most often made by either a neurologist or a geriatrician working in a primary care memory clinic or in specialized hospital clinic. The clinical diagnostic process includes brain imaging with computer tomography or MRI. Cerebrospinal fluid biomarkers and/or PET-imaging (amyloid-PET or FDG-PET) is used in differential diagnostics if needed, less often in patients who are over 75 years at time of diagnosis. The diagnosis is based the clinical examination combined with symptoms, interviewing the patient and a close relative/ friend/ care giver and typical structural findings on MRI/CT (hippocampal atrophy in AD, vascular changes in vascular dementia (VaD)) [31]. The diagnosis is set according to the ICD-10. The diagnostic criteria for AD and VaD have not changed during the follow-up of the study.

### 2.5 *The CAIDE risk score*

The CAIDE risk score predicts the risk of developing dementia within 20 years among middle aged people by classifying an individual into one of the five risk categories where the lowest risk category represents a 1.0 % risk for dementia during 20 years and the highest risk category a 16.4 % risk***.*** The development of the CAIDE risk score has been previously described [9]. CAIDE risk score was used as described in the original article*,* i.e. the CAIDE risk score variables were classified according to the cut-offs of the CAIDE risk score and weighted risk factors were summed to provide a total score. In concord with the original CAIDE article, we included age, education, sex, systolic blood pressure, BMI, total cholesterol and physical activity into CAIDE Model 1. In CAIDE Model 2 *APOE* ε4 genotype status was added into the risk score (*APOE* ε4 non-carriers received 0 points and *APOE* ε4 carriers 2 points, as described in the original CAIDE article) and the risk score variables age (>53 years 5 points instead of 4 points in CAIDE Model 1) and education (7–9 years 3 points instead of 2 points in CAIDE Model 1; 0–6 years 4 points instead of 3 points in CAIDE Model 1) were weighted in a slightly different way than in CAIDE Model 1 [9].

### 2.6 *Statistical analysis*

We computed the CAIDE risk scores for each participant exactly as previously described [9] for CAIDE Models 1 and 2. For Model 2 the study population was slightly smaller than for Model 1, since Model 2 included *APOE* genotype which was known for 95.9 % of the participants. No imputations for missing data were performed. After that we substituted BMI with insulin resistance measured by HOMA-IR in both CAIDE Models. There is no validated cut-off for normal HOMA-IR and the distribution of HOMA-IR is very skewed, which is why HOMA-IR values were divided into “low” HOMA-IR and “high” HOMA-IR according to the median HOMA-IR (1.63) of the study population. HOMA-IR was weighted in the CAIDE Models as BMI; HOMA-IR ≤ 1.63 was scored with 0 point (BMI ≤ 30 kg/m²) and HOMA-IR > 1.63 was scored with 2 points (BMI > 30 kg/m²).

Kaplan-Meier estimates were calculated for time for dementia diagnosis and separately for time for AD diagnosis according to CAIDE sum score. Death of another cause than dementia during follow-up resulted in the participant being censored from the analyses. The accuracy of the CAIDE risk score to predict 19-year risk of all-cause dementia and AD separately was quantified by calculating the ROC (receiver-operating characteristics curve) area under the curve (AUC) and 95 % confidence intervals calculated with logistic regression. The maximum AUC with a value 1.0 means that the test is perfect in the differentiation between the diseased and non-diseased, and AUC 0.5 means same as chance [32].

For all numerical variables, normality assumptions were evaluated visually using Q-Q plots (normal quantile plot), box-plot, kurtosis and skewness evaluation. To analyze the differences between study characteristics we used Student's two-sample *t*-test for variables with a normal distribution, the Wilcoxon rank sum test otherwise, and ChiSquare test for categorical variables. Differences in characteristics among the risk score groups were analyzed with analysis of variance (ANOVA) or Kruskal-Wallis test.

Statistical significance was set at *p* < 0.05 (two-sided) for all analyses. The analyses were carried out using JMP®, Version 17.0 (Pro SAS Institute Inc., Cary, NC, 1989–2019). ROC AUC confidence intervals were analyzed with SAS Software, Version 9.4 of the SAS System for Windows (SAS Institute Inc., Cary, NC, USA).

## 3 Results

### 3.1 *Study population*

Our study population included 5806 participants, with a mean age of 52.1 years (SD 14.5), range 30–97. During the follow-up (mean 19.2 years, SD 0.12), dementia was diagnosed in 571 (9.8 %) participants and in 451 of them (79.0 %) the diagnosis was AD. The mean age of dementia diagnosis was 69.5 years (SD 12.1), (Q1-Q3 59.5–78.7 years). The mean time between examination and dementia outcome was 10.6 years (SD 5.2). 1434 (24.7 %) participants died during the follow-up and 374 of the deceased participants were diagnosed with dementia during the study. The *APOE* ε4 genotype status was known for 5566 (95.9 %) participants.

The characteristics of the study population according to dementia status are shown in Table 1. The participants who developed dementia were significantly older, less educated and more often women than those who remained without dementia (all *p* < 0.0001). Individuals who developed dementia had more often hypertension or elevated systolic blood pressure and diabetes but they were less often smokers than those who remained without dementia (all *p* < 0.0001) in our study population. Developing dementia was also associated with higher BMI, total cholesterol, LDL, *APOE* ε4 carriership and higher CAIDE score (all <0.001). There was no difference in HOMA-IR values between those with and without dementia. Of note, the age difference between those with and without dementia was large and many of the aforementioned risk factors associate with older age and may thus be associated with ageing itself rather than dementia risk specifically.

**Table 1:** Characteristics of the study population at baseline according to dementia status during the 19-year follow-up.

	Without Dementia (*n* = 5235)	With Dementia (*n* = 571)	p
Age, years	50.2 (13.6)	70.0 (9.7)	<0.0001
Education, years	11.7 (4.0)	8.2 (3.4)	<0.0001
Men, n (%)	2419 (46 %)	208 (36 %)	<0.0001
BMI, kg/m²	26.8 (4.7)	27.5 (4.3)	0.0003
SBP, mmHg	132.9 (20.4)	147.5 (22.1)	<0.0001
Hypertension, n (%)	2320 (44 %)	406 (71 %)	<0.0001
Total cholesterol, mmol/L	5.9 (1.1)	6.2 (1.1)	<0.0001
LDL, mmol/L	3.7 (1.1)	3.9 (1.1)	<0.0001
HDL, mmol/L	1.3 (0.4)	1.4 (0.4)	0.31
Triglycerides, mml/L (IQR)	1.3 (1–1.8)	1.4 (1.1–2)	0.07
HOMA-IR (IQR)	1.6 (1.1–2.5)	1.8 (1.2–2.8)	0.19
Diabetes, n (%)	180 (3 %)	50 (9 %)	<0.0001
Smokers, n (%)	1492 (29 %)	48 (8 %)	<0.0001
Physically inactive, n (%)	2149 (41 %)	218 (38 %)	0.18
*APOE* ε4 carriers, %	1508 (30 %)	238 (43 %)	<0.0001
CAIDE risk score, points (IQR)	5 (2–8)	9 (7–11)	<0.0001

The results are shown as mean (SD) or median (interquartile range) unless stated otherwise. *p*-value indicates overall differences between groups. Participants with missing values (LDL: 27 participants from without dementia group and 3 from with dementia group, smokers: 1 + 2, *APOE* ε4: 217+23) were excluded from the corresponding analyses.

BMI = body mass index, SBP = systolic blood pressure, HOMA-IR = homeostatic model assessment for insulin resistance, physically inactive = leisure time physical activity less often than twice a week lasting at least 30 min and causing at least mild breathlessness and sweating.

Characteristics of the study population divided into CAIDE risk score categories (CAIDE Model 1) are shown in Table 2. The higher CAIDE risk score categories were associated with older age, less education in years, and higher systolic blood pressure, BMI, HOMA-IR, total cholesterol, LDL cholesterol and triglycerides, and lower HDL cholesterol (*p* < 0.0001 for overall differences among the CAIDE risk score categories).

**Table 2:** Characteristics of the study population according to CAIDE risk score categories.

	CAIDE risk score categories					
	0–5 p.	6–7 p.	8–9 p.	10–11 p.	12–15 p.	p-value
Age, years (IQR)	40 (35–46)	54 (49–63)	61 (54–70)	65 (58–74)	69 (61–76)	<0.0001
Education, years	13.6 (3.4)	11.2 (3.5)	9.2 (3.3)	7.6 (2.5)	6.3 (2.0)	<0.0001
Systolic BP, mmHg	122.7 (13.9)	135.6 (18.2)	145.0 (19.7)	153.8 (19.3)	159.0 (18.9)	<0.0001
BMI, kg/m2	25.4 (4.1)	27.1 (4.2)	27.8 (4.6)	29.2 (4.8)	31.5 (4.6)	<0.0001
HOMA-IR (IQR)	1.36 (0.9–2)	1.6 (1.1–2.6)	1.9 (1.3–2.9)	2.2 (1.4–3.6)	3.0 (1.8–4.6)	<0.0001
Total cholesterol, mmol/L	5.6 (1.0)	6.0 (1.0)	6.2 (1.1)	6.5 (1.2)	6.9 (1.1)	<0.0001
LDL, mmol/L	3.4 (0.9)	3.8 (1.0)	3.9 (1.0)	4.2 (1.1)	4.6 (1.1)	<0.0001
HDL, mml/L	1.4 (0.4)	1.3 (0.4)	1.3 (0.4)	1.3 (0.4)	1.2 (0.3)	<0.0001
Triglycerides, mmol/L (IQR)	1.1 (0.9–1.6)	1.3 (1–1.8)	1.4 (1.1–2)	1.6 (1.2–2.2)	1.8 (1.4–2.5)	<0.0001

Systolic BP = systolic blood pressure, BMI = body mass index, HOMA-IR = homeostatic model assessment for insulin resistance. The results are shown as mean (SD) or median (interquartile range). *p*-value indicates overall differences between groups, assessed with Kruskal-Wallis test.

### 3.2 *CAIDE dementia risk score and incident dementia*

Formation of the CAIDE risk score within different risk score categories is demonstrated in Appendix Table A.1. In the highest risk score category, our study participants received more points from all of the variables. Majority of the participants did not obtain points from the BMI variable in different risk score categories with the exception of the highest risk score category.

**Fig. 2:**
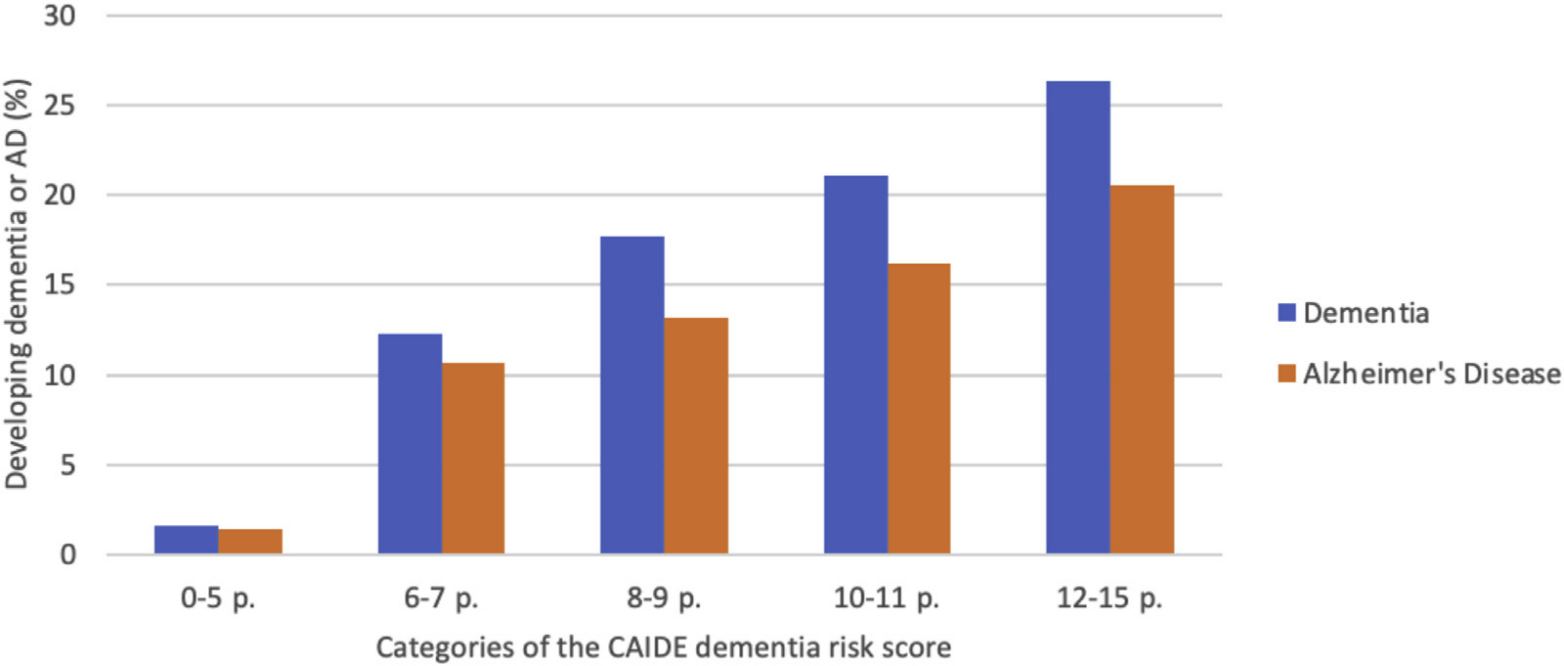
The probability of developing dementia or Alzheimer's disease in 19 years follow-up according to the categories of the CAIDE dementia risk score.

Fig. 2 shows the probability of developing dementia and Alzheimer's disease according to the categories of the CAIDE risk score. Higher CAIDE score was associated with higher overall risk for dementia and with a higher risk for AD during the 19-year follow-up: 1.6 % risk of dementia in the lowest CAIDE risk category vs. 26.3 % risk in the highest category (Fig. 2).

### 3.3 *Surviving without dementia according to CAIDE risk score categories*

The accuracy and hazard ratios of the CAIDE risk score for predicting dementia were calculated in our study population. The AUC for CAIDE Model 1 for predicting dementia was 0.78 (95 % Cl 0.76–0.79) and for CAIDE Model 1 with HOMA-IR instead of BMI the AUC was 0.78 (95 % Cl 0.76–0.80). In CAIDE Model 2 the AUC values increased compared to CAIDE Model 1: the AUC for CAIDE Model 2 was 0.81 (95 % Cl 0.80–0.83) and for CAIDE Model 2 with HOMA-IR instead of BMI the AUC was 0.81 (95 % Cl 0.80–0.83).

As Alzheimer's disease was the cause of dementia in the majority of all dementia cases, the AUC for predicting Alzheimer's disease was almost the same as for overall dementia. The AUC for CAIDE Model 1 for predicting AD was 0.76 (95 % Cl 0.75–0.78) for CAIDE Model 1 with HOMA-IR instead of BMI the AUC was 0.76 (95 % Cl 0.75–0.78). In CAIDE Model 2 the AUC was 0.80 (95 % Cl 0.79–0.82), and for CAIDE Model 2 with HOMA-IR instead of BMI the AUC was 0.81 (95 % Cl 0.79–0.82).

## 4 Discussion

**Fig. 3:**
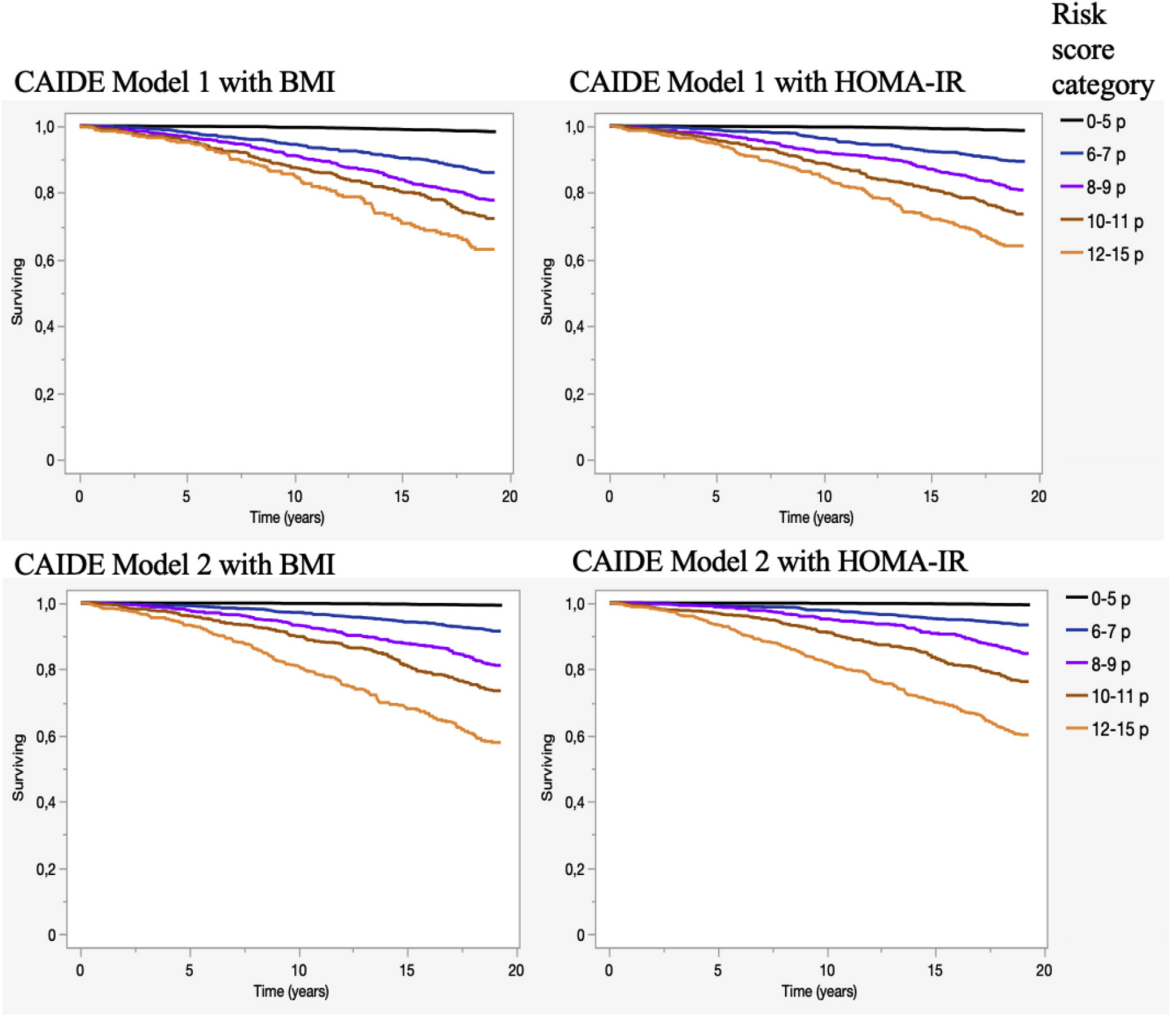
Surviving without dementia according to different CAIDE dementia risk score categories. CAIDE Model 1 included age, education, sex, systolic blood pressure, BMI, total cholesterol and physical activity. In CAIDE Model 2 *APOE* ε4 genotype status was added into the CAIDE risk score and risk score variables on age and education were weighted in a slightly different way than CAIDE Model 1. In both CAIDE Models we used two different methods to calculate the risk score, first by using the previously published CAIDE risk score and then by substituting BMI with insulin resistance measured by HOMA-IR. Surviving = Overall survival without dementia diagnosis during 19 years follow-up.

Here, we demonstrate that the CAIDE risk score has good accuracy for predicting both overall dementia and Alzheimer´s dementia in a representative sample of the Finnish adult population aged over 30 years. Accordingly, the higher the CAIDE risk score category, the higher the risk of developing dementia during a 19-year follow-up (1.6 % in the lowest category vs. 26.3 % risk of dementia in the highest category). The probability of surviving without dementia decreased systematically as CAIDE risk score category increased as presented in the Kaplan-Meier analyses (Fig. 3). Moreover, adding *APOE* ε4 genotype to the risk score improved the prediction power of the CAIDE dementia risk score in our study, and a similar finding has been shown earlier in a clinical sample [33]. Secondly, the results remained unchanged when replacing the obesity variable with insulin resistance measured by HOMA-IR as part of the CAIDE risk score.

The CAIDE dementia risk score presented a good accuracy to predict dementia in a Finnish population-based cohort - which was a representative sample of the Finnish adult population - during 2000 to 2019. The CAIDE risk score was developed by utilizing a population-based cohort from Eastern Finland and data for the original CAIDE risk score was gathered during the years 1972–1987 when the participants were aged from 39 to 64 years. Although both the Health 2000 study and the CAIDE study are based on a Finnish cohort there are some notable differences between the studies: in the CAIDE study the dementia diagnoses were based on a thorough clinical examination and included neuroimaging as well as evaluation by an expert panel. In contrast, the dementia diagnoses of the present study are based on register data from Finnish national registries. Therefore, the results of the present study expand the results of the CAIDE study and demonstrate that the CAIDE risk score can be used to predict dementia diagnosis in clinical practice, without a specialist diagnostic setting. Previously, dementia diagnoses derived from national registries in Finland have been found to be very accurate, although there might be a risk for underestimation of dementia [34]. Therefore, our results are likely to underestimate, rather than overestimate the incidence of dementia during the follow-up.

The association of the CAIDE risk score with neuroradiological findings provide further evidence for the utility of this screening instrument: Higher midlife CAIDE scores have been shown to associate with white matter hyperintensities after 20 years and medial temporal lobe atrophy after 30 years follow-up [20]. Moreover, in elderly persons, higher CAIDE risk score has been associated with an increased risk of cerebral infarcts [15]. However, the CAIDE risk score has not been found to be associated with amyloid accumulation in a positron emission tomography (PET) study [19]. Lastly, the CAIDE score has also been examined in association with neuropsychological tests: higher CAIDE scores associated with lower performance in executive function and visual perception and construction [35]. These findings suggest that CAIDE could predict cognitive impairment in the domains that are the main characteristics of vascular cognitive impairment [18].

In our study the CAIDE dementia risk score values obtained by using HOMA-IR and BMI were similar. Insulin resistance has been shown to associate with cognitive decline and dementia [21,36]. It has also been shown that higher midlife, but not late-life HOMA-IR associates with cerebrocortical Aβ load in late-life [21,23]. Findings on the association of BMI with dementia have been mixed: Higher BMI seems to be associated with either an increased or a decreased risk of dementia depending on the length of follow-up [37]. Moreover, BMI and cholesterol measured in late-life have not been associated with increased risk of AD in previous research [38,39]. Although CAIDE measures hyperlipidemia based on cholesterol levels, recent studies have highlighted the potential value of ApoB in more accurately assessing risk [40]. However, HOMA-IR and BMI are closely associated and BMI is easier and more practical to measure than HOMA-IR. In addition, there is no established cut-off for HOMA-IR which makes it difficult to utilize HOMA-IR in the clinic at an individual level. According to our results, there is no added value in measuring HOMA-IR instead of BMI to predict future dementia risk.

We found the CAIDE risk score to be accurate for predicting dementia and Alzheimer's disease despite the broad age range in our cohort. However, in previous research the CAIDE dementia risk score's accuracy in older adults with short follow-up time [13,16] and in Japanese-American men has been found to be limited [14]. As age represents accumulative sum of risk factors it might explain why the predictive accuracy dementia risk models has been thought to be poor in individuals aged 80 years or older [16]. Also, many studies have shown that the association between vascular risk factors and dementia risk is reversed close to the onset of dementia [41,42]. A plausible explanation for this paradox is that the prodromal symptoms of dementia often lead to weight loss [43] and a decline in cholesterol values [44].

This study has the following strengths: Data from a large nationally representative population-based cohort and a long follow-up of 19 years enhances the generalizability of the results. As the cohort was based on the Health 2000 Study, systematically collected variables were available for analysis. Additionally, as we used register-based data for dementia diagnosis, no participants were lost during follow-up. Also, the diagnostic process for the clinical diagnosis of dementia in Finland typically includes neuroimaging and is guided by nationally available care guidelines [31] which makes the diagnoses reliable. The diagnostic criteria for AD and vascular dementia did not change during the follow-up. Lastly, the data for this study was gathered before the COVID-19 pandemic, which would have otherwise presented as a potential source of bias.

There are also a few limitations: Health 2000 Survey is a representative population-based cross-sectional study and the participants have been of different ages (range 30–97 years) at the time of collecting the data. The CAIDE risk score includes age, which is the strongest risk factor for dementia and the age variable has a strong weighting in the CAIDE Models. Also, the treatment practices of dyslipidemia and hypertension have changed over the years: thus, variables could be changed during the follow-up. Individuals with baseline dementia diagnosis were excluded, but it is probable that the population included individuals with mild cognitive impairment. The data of dementia diagnosis made in outpatient primary care (The Care Notification Register) was unavailable for analysis in those cases where the participant was not hospitalized or did not die or did not reimburse medications for memory disorders during follow-up. However, this source of bias is more likely to attenuate than to inflate the associations observed. Deaths due to other causes than dementia were censored in the analyses which is again likely to dilute the results, since those who died of other causes might have developed dementia if they would have lived longer. Also, the CAIDE risk score does not account for all known modifiable risk factors for dementia, such as hearing loss, traumatic brain injury, excessive alcohol consumption, social isolation and depression [45]. It is important to screen for and treat these risk factors in the clinic, and it might be useful to develop new predictive risk scores in the future including these risk factors to predict dementia more accurately.

In conclusion, the CAIDE dementia risk score predicts dementia well in a national population-based cohort. The predictive value of insulin resistance measured by HOMA-IR is comparable to that of obesity as part of the CAIDE risk score but does not offer additional value to predict dementia risk. Nevertheless, it seems plausible that interventions targeted at insulin resistance in midlife would be most effective in individuals with high HOMA-IR. Of note, treating obesity is the most efficient means of treating insulin resistance, since abdominal obesity is an important cause of insulin resistance [46]. Whether interventions aimed at treating insulin resistance and/or obesity would lower dementia risk remains to shown. These findings imply that the CAIDE risk score is applicable for assessing risk of dementia between high- and low-risk individuals at population level, and highlights the importance of interventions targeted at modifiable risk factors of dementia.

## Funding

EP was supported by the Finnish Governmental Research Funding (VTR) for Turku University Hospital and the Turku University Foundation. LLE was supported by the Paulo Foundation, the Finnish Medical Foundation, and by the Finnish Governmental Research Funding (VTR) for Turku University Hospital.

## CRediT authorship contribution statement

**Elina Pietilä:** Writing – review & editing, Writing – original draft, Visualization, Methodology, Funding acquisition, Formal analysis, Conceptualization. **Eliisa Löyttyniemi:** Writing – review & editing, Supervision, Software, Methodology, Formal analysis, Data curation, Conceptualization. **Seppo Koskinen:** Writing – review & editing, Data curation, Conceptualization. **Jenni Lehtisalo:** Writing – review & editing, Data curation, Conceptualization. **Matti Viitanen:** Writing – review & editing, Supervision, Methodology, Funding acquisition, Conceptualization. **Juha O. Rinne:** Writing – review & editing, Supervision, Methodology, Funding acquisition, Conceptualization. **Antti Jula:** Writing – review & editing, Supervision, Methodology, Data curation, Conceptualization. **Laura L. Ekblad:** Writing – review & editing, Writing – original draft, Visualization, Supervision, Project administration, Methodology, Funding acquisition, Formal analysis, Conceptualization.

## Declaration of competing interest

The authors declare that they have no known competing financial interests or personal relationships that could have appeared to influence the work reported in this paper.

## Figures and Tables

**Table A1:** Formation of CAIDE dementia risk score within different risk categories (CAIDE Model 1).

	CAIDE	Risk score categories					
	risk score	0–5 p.	6–7 p.	8–9 p.	10–11 p.	12–15 p.	p-value
Age							<0.0001
< 47 years	0	2195 (77 %)	98 (10 %)	20 (2 %)	0 (0 %)	0 (0 %)	
47–53 years	3	441 (16 %)	349 (36 %)	219 (22 %)	84 (13 %)	13 (4 %)	
> 53 years	4	198 (7 %)	534 (54 %)	753 (76 %)	588 (88 %)	314 (96 %)	
Education							<0.0001
≥ 10 years	0	2616 (92 %)	612 (62 %)	302 (30 %)	77 (11 %)	4 (1 %)	
7–9 years	2	211 (7 %)	338 (34 %)	543 (55 %)	383 (57 %)	147 (45 %)	
0–6 years	3	7 (0 %)	31 (3 %)	147 (15 %)	212 (32 %)	176 (54 %)	
Sex							<0.0001
Women	0	1658 (59 %)	488 (50 %)	517 (52 %)	356 (53 %)	160 (49 %)	
Men	1	1176 (41 %)	493 (50 %)	475 (48 %)	316 (47 %)	167 (51 %)	
Systolic BP							<0.0001
≤ 140 mmHg	0	2621 (92 %)	661 (67 %)	435 (44 %)	137 (20 %)	27 (8 %)	
> 140 mmHg	2	213 (8 %)	320 (33 %)	557 (56 %)	535 (80 %)	300 (92 %)	
BMI							<0.0001
≤ 30 kg/m²	0	2538 (90 %)	795 (81 %)	730 (74 %)	377 (56 %)	87 (27 %)	
> 30 kg/m²	2	296 (10 %)	186 (19 %)	262 (26 %)	295 (44 %)	240 (73 %)	
Total cholesterol							<0.0001
≤ 6.5 mmol/L	0	2478 (87 %)	705 (72 %)	635 (64 %)	327 (49 %)	78 (24 %)	
> 6.5 mmol/L	2	356 (13 %)	276 (28 %)	357 (36 %)	345 (51 %)	249 (76 %)	
Physical activity							<0.0001
Active	0	1761 (62 %)	606 (62 %)	613 (62 %)	355 (53 %)	104 (32 %)	
Inactive	1	1073 (38 %)	375 (38 %)	379 (38 %)	317 (47 %)	223 (68 %)	
	Total	*n* = 2834	*n* = 981	*n* = 992	*n* = 672	*n* = 327	
	Dementia	46 (1.6 %)	121 (12.3 %)	176 (17.7 %)	142 (21.1 %)	86 (26.3 %)	
	AD	39 (1.4 %)	105 (10.7 %)	131 (13.2 %)	109 (16.2 %)	67 (20.5 %)	

The CAIDE risk score variables were classified into different risk scores of which weightings by risk score points are shown in the second column. The participants’ distribution for each variable within risk score category is shown (n/ %). The proportion of Alzheimer's disease among the dementia in each category is shown. p-value indicates overall differences between groups.

BMI = body mass index, Systolic BP = systolic blood pressure, inactive = leisure time physical activity 30 min while at least mildly breathing less often than twice a week. AD = Alzheimer's disease.
