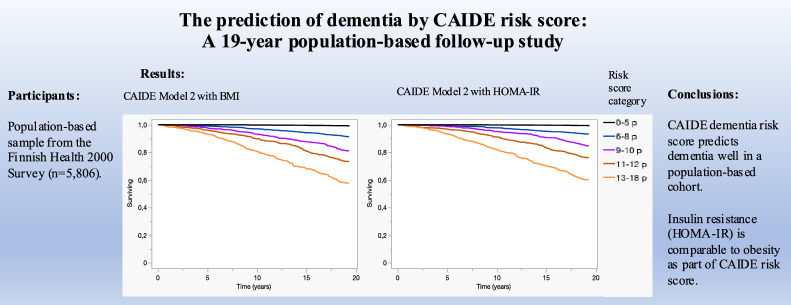# Corrigendum to Enhancing dementia prediction: A 19-year validation of the CAIDE risk score with insulin resistance and APOE ε4 integration in a population-based cohort

**DOI:** 10.1016/j.tjpad.2025.100158

**Published:** 2025-04-02

**Authors:** Elina Pietilä, Eliisa Löyttyniemi, Seppo Koskinen, Jenni Lehtisalo, Matti Viitanen, Juha O. Rinne, Antti Jula, Laura L. Ekblad

**Affiliations:** a Turku PET Centre, University of Turku, Turku, Finland; b Turku PET Centre, Turku University Hospital, Turku, Finland; c Department of Biostatistics, University of Turku and Turku University Hospital, Finland; d Finnish Institute for Health and Welfare, Helsinki, Finland; e Department of Geriatrics, Turku University Hospital, Wellbeing services county of Southwestern Finland, Finland; f Division of Clinical Geriatrics, NVS, Karolinska Institutet, Stockholm, Sweden; g InFLAMES Research Flagship Center, University of Turku, Turku, Finland; h Finnish Institute for Health and Welfare, Turku, Finland

The authors regret that we noticed a small error in the legend of the Fig. 3. In the figure legend the thresholds of the risk score categories should have been different for Model 2 than for Model 1. Attached is an image where the thresholds of these categories for Model 2 have been corrected in the legend. The thresholds of these categories for Model 2 are also corrected in the legend of the graphical abstract. The authors would like to apologise for any inconvenience caused.

**Fig. 3:**
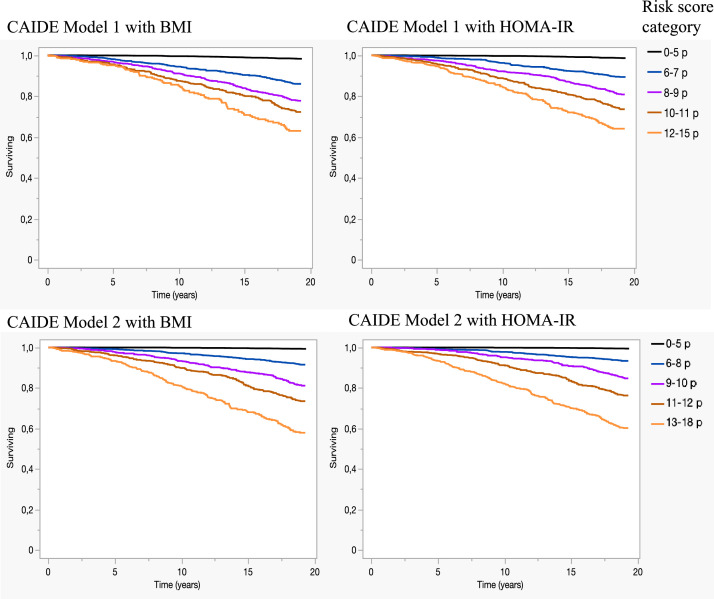
Surviving without dementia according to different CAIDE dementia risk score categories.

**Figure:**